# A dynamic, embodied paradigm to investigate the role of serotonin in decision-making

**DOI:** 10.3389/fnint.2013.00078

**Published:** 2013-11-21

**Authors:** Derrik E. Asher, Alexis B. Craig, Andrew Zaldivar, Alyssa A. Brewer, Jeffrey L. Krichmar

**Affiliations:** ^1^Cognitive Anteater Robotics Lab, Department of Cognitive Sciences, University of CaliforniaIrvine, CA, USA; ^2^Laboratory of Visual Neuroscience, Department of Cognitive Sciences, University of CaliforniaIrvine, CA, USA; ^3^Cognitive Anteater Robotics Lab, Department of Computer Science, University of CaliforniaIrvine, CA, USA

**Keywords:** serotonin, embodiment, cost assessment, human–robot interaction, adaptive agents, game theory, cognitive modeling, acute tryptophan depletion

## Abstract

Serotonin (5-HT) is a neuromodulator that has been attributed to cost assessment and harm aversion. In this review, we look at the role 5-HT plays in making decisions when subjects are faced with potential harmful or costly outcomes. We review approaches for examining the serotonergic system in decision-making. We introduce our group’s paradigm used to investigate how 5-HT affects decision-making. In particular, our paradigm combines techniques from computational neuroscience, socioeconomic game theory, human–robot interaction, and Bayesian statistics. We will highlight key findings from our previous studies utilizing this paradigm, which helped expand our understanding of 5-HT’s effect on decision-making in relation to cost assessment. Lastly, we propose a cyclic multidisciplinary approach that may aid in addressing the complexity of exploring 5-HT and decision-making by iteratively updating our assumptions and models of the serotonergic system through exhaustive experimentation.

## INTRODUCTION

Recent theoretical work has implicated serotonergic (5-HT) function in critical dimensions of reward versus punishment and invigoration versus inhibition (Boureau and Dayan, 2010). Both of these dimensions have influence on a broad range of decision-making elements, including reward processing, impulsivity, reward discounting, predicting punishment, harm aversion, opponency with other neuromodulators, and anxious states (Doya, 2008; Dayan and Huys, 2009; Cools et al., 2010). In this review, we first provide evidence from the literature indicating several of the proposed functions attributed to the serotonergic system. Next, we discuss approaches that utilized game theory and other behavioral measures along with some metric of serotonergic function. Finally, we introduce a multidisciplinary experimental paradigm to model the role 5-HT plays in decision-making, and present some of our work that has utilized this paradigm.

Our paradigm begins with base assumptions regarding the role of serotonin or other neuromodulators to construct a simulated agent that can adapt to environmental challenges. That adaptive agent, which is either embodied in a robotic platform for human–robot interaction studies or embedded in a computer interface, is incorporated into a game theoretic environment. The data collected from these experiments are then analyzed to support or reject hypotheses about the roles of neuromodulators in specific cognitive functions such as decision-making, which may lead to the use of more sophisticated adaptive agents in subsequent studies.

### FUNCTIONAL ROLES OF SEROTONIN

It has been suggested that serotonin influences a broad range of decision-based functions such as reward assessment, cost assessment, impulsivity, harm aversion, and anxious states. This section discusses recent evidence demonstrating the role serotonin has on these decision-based functions.

Though reward processing is a function that has primarily been attributed to the dopaminergic (DA) system, 5-HT has also been associated with reward-related behavior (Tanaka et al., 2007, 2009; Nakamura et al., 2008; Schweighofer et al., 2008; Bromberg-Martin et al., 2010; Okada et al., 2011; Seymour et al., 2012). Recent single-unit recordings of serotonergic neurons in the monkey dorsal raphe nucleus (DRN), which is a major source of serotonergic innervation in the central nervous system, demonstrated that many of these neurons represent reward information (Nakamura et al., 2008; Bromberg-Martin et al., 2010; Okada et al., 2011). Nakamura et al. (2008) showed that during a saccade task, after target onset but before reward delivery, the activity of many DRN neurons was modulated by the expected reward size. Bromberg-Martin et al. (2010) showed that a group of DRN neurons tracked progress toward future delayed reward after the initiation of a saccade and after the value of the trial was revealed. These studies suggest that DRN neurons, which include 5-HT neurons, may influence behavior based on the amount of delay before reward delivery and the value of the reward in future motivational outcomes (Nakamura et al., 2008; Bromberg-Martin et al., 2010).

Other anatomical evidence has shown that projections from DRN to reward-related DA regions support 5-HT’s role in both reward and punishment (Tops et al., 2009). A theoretical review by Boureau and Dayan (2010) suggested that the 5-HT and dopamine systems primarily activate in opposition and at times in collaboration for goal directed actions. A review by Doya (2008) also highlighted possible computational factors of decision-making in brain regions innervated by serotonin and dopamine (for a schematic of the potential interplay between 5-HT and other brain structures, see Doya, 2008, Figure 3). 5-HT projections to dopamine areas have been shown to regulate threat avoidance (Rudebeck et al., 2006; Tops et al., 2009), and an impairment in these projections can lead to impulsivity and addiction (Deakin, 2003). Altogether, the interaction between these systems allows 5-HT to play various functional roles in decision-making where reward and punishment, as well as invigoration and inhibition, are in opposition.

In addition to reward processing, several studies have investigated serotonin’s involvement in reward and impulsivity by manipulating levels of central 5-HT in humans using the acute tryptophan depletion (ATD) procedure. ATD is a dietary reduction of tryptophan, an amino acid precursor of 5-HT, which causes a rapid decrease in the synthesis and release of the human brain’s central 5-HT, thus affecting behavioral control (Nishizawa et al., 1997). Altering 5-HT levels via ATD influences a subject’s ability to resist a small immediate reward over a larger delayed reward (delay reward discounting; Tanaka et al., 2007, 2009; Schweighofer et al., 2008). As such, subjects that underwent ATD had both an attenuated assessment of delayed reward and a bias toward small reward, which were indicative of impulsive behavior.

Besides reward, 5-HT has also been linked to predicting punishment or harm aversion (Cools et al., 2008; Crockett et al., 2009, 2012; Tanaka et al., 2009; Seymour et al., 2012). Cools et al. (2008) paired the ATD procedure with a reversal-learning task, demonstrating that subjects under ATD made more prediction errors for punishment-associated stimuli than for reward-associated stimuli. In a related study, Crockett et al. (2009) utilized the ATD procedure with a Go/No-Go task to show that lowering 5-HT levels resulted in a decrease in punishment-induced inhibition. In a follow up study, they investigated the mechanisms through which 5-HT regulated punishment-induced inhibition by using the ATD procedure paired with their Reinforced Categorization task, a variation on the Go/No-Go task (Crockett et al., 2012). Subjects with lowered 5-HT were faster in responding to stimuli predictive of punishments (Crockett et al., 2012), indicating a manipulation of some punishment-predicting mechanism associated with standard serotonergic function. Together, these results suggest that 5-HT influences the ability to inhibit actions that predict punishment and to avoid harmful circumstances.

Beyond punishment, 5-HT has been implicated in stress and anxiety (Millan, 2003; Jasinska et al., 2012). A recent review by Jasinska et al. (2012) proposed a mechanistic model between environmental impact factors and genetic variation of the serotonin transporter (5-HTTLPR), linking to the risk of depression in humans. They argued that genetic variation may be linked to a balance in the brain’s circuitry underlying stressor reactivity and emotion regulation triggered by a stressful event, ultimately leading to depression (Jasinska et al., 2012). A review by Millan (2003) described studies showing that 5-HT function has been tied to an organism’s anxious states triggered by conditioned or unconditioned fear. Together, this work suggests a functional role for 5-HT in the control of anxious states.

In summary, these studies reveal serotonergic modulation of a wide range of decision-based functions including but not limited to reward processing, motivational encoding, punishment prediction, discounting, impulsivity, harm aversion, and anxious states. Building on this body of work, many researchers in the field have utilized their own approaches in studies to better understand the function of serotonin in behavior. In the present paper, we introduce a novel, multi-disciplinary approach to study serotonin’s influence in decision-making that may highlight many of the functions described above. Our paradigm combines techniques from computational neuroscience, socioeconomic game theory, human–robot interaction, and Bayesian statistics.

### INVESTIGATION OF DECISION-MAKING USING GAME THEORY AND SEROTONERGIC MANIPULATIONS

Game theory is a toolbox that is utilized in a multitude of disciplines for its ability to quantitatively measure and predict behavior in situations of cooperation and competition (Maynard Smith, 1982; Nowak et al., 2000; Skyrms, 2001). It operates on the principle that organisms will balance reward with effort while acting in self-interest to obtain the optimal result in a given situation. Game theory is especially valuable as a venue for studying human behavior because it provides a replicable, predictable, and controlled environment with clearly defined boundaries. These elements are essential when introducing computer agents as opponents.

Game theory has been combined with manipulations of serotonin to help understand its role in socioeconomic decision-making. For example, in the Prisoner’s Dilemma, where subjects either cooperate or defect in a risky situation, it has been shown that ATD increases the prevalence of defecting, which might be considered an impulsive, risk-taking choice (Wood et al., 2006). Similarly, the Ultimatum game is a test of cooperation in which a proposer offers a share of a resource to a receiver, and the receiver can either accept or reject this offer (Nowak et al., 2000; Sanfey, 2003). In studies conducted by Crockett et al. (2008) incorporating the Ultimatum game with serotonergic manipulations, it was found that subjects under ATD rejected a significantly higher proportion of unfair offers and that decreased serotonin levels correlated with increased dorsal striatal activity induced by costly punishment (Crockett et al., 2013). In contrast, subjects that ingested citalopram, an SSRI, were less likely to punish unfairness in the Ultimatum game (Crockett et al., 2010). Together, these studies implicate the involvement of the serotonergic system with cost in decision-making, an important result in understanding the cost and reward mechanisms in the brain.

Another notable game that focuses on the investigation of cooperation and social contracts is the Stag Hunt. In the Stag Hunt, two players must independently choose to hunt a high payoff stag cooperatively or a low payoff hare individually. The risk in decision-making lies in the case when only one player chooses stag, resulting in no payoff for that player (Skyrms, 2004). The body of work involving Stag Hunt largely involves simulations with set-strategy agents or human players as opponents (Skyrms, 2004; Szolnoki and Perc, 2008; Scholz and Whiteman, 2010). More recently, the use of adaptive agents, computer players that learn in real-time, have been gaining popularity in the field of social decision-making (Yoshida et al., 2008, 2010). Yoshida et al. (2010) conducted a study in which adaptive agents played a spatiotemporal version of the Stag Hunt game against human subjects in an fMRI scanner, implicating both rostral medial prefrontal cortex and dorsolateral prefrontal cortex in processing uncertainty and sophistication of agent strategy, respectively. Utilizing adaptive agents allows for a dynamic yet controlled behavioral manipulation in subjects, which is useful within game environments, particularly if applied to studying the cost and reward mechanisms of the brain.

Pairing a decision-making task with ATD in the absence of game theory has further illuminated serotonin’s involvement in behavior. This combination has revealed serotonin’s involvement in the reflexive avoidance of relatively immediate small costs in favor of larger future costs with an Information Sampling Task (Crockett et al., 2011). The pairing of ATD with a “four-armed bandit” task showed that depleted subjects tended to be both more perseverative and less receptive to reward (Seymour et al., 2012). These results show that the combination of a decision-making task with serotonergic manipulation (e.g., ATD) can provide important information about the role serotonin has in the decision-making process. In general, the combination of ATD with a decision-making task provides a useful venue for the exploration of social behavior and the neural correlates of cost and reward in decision-making.

In addition to altering decision-making, reduced 5-HT levels via ATD have been correlated with individual differences in subject behavior (Krämer et al., 2011; Demoto et al., 2012). Subjects with high neuroticism and low self-directedness personality traits have been shown to be particularly susceptible to central 5-HT depletion, resulting in decreased selection of delayed larger reward over smaller immediate reward when performing a delayed reward choice task (Demoto et al., 2012). Similarly, subjects with low baseline aggression have displayed reduced reactive aggression when performing a competitive reaction time task with depleted 5-HT levels (Krämer et al., 2011). The results from these studies provide evidence for individual behavioral differences correlated with central 5-HT manipulation, which may serve as a direction for future study.

In summary, due to the complex nature of the serotonergic system, researchers have utilized several complementary methods to investigate the varied aspects of its behavioral influence. This review introduces a multidisciplinary experimental paradigm to model the role 5-HT plays in decision-making.

## A MULTIDISCIPLINARY PARADIGM TO INVESTIGATE THE SEROTONERGIC SYSTEM

**FIGURE 1:**
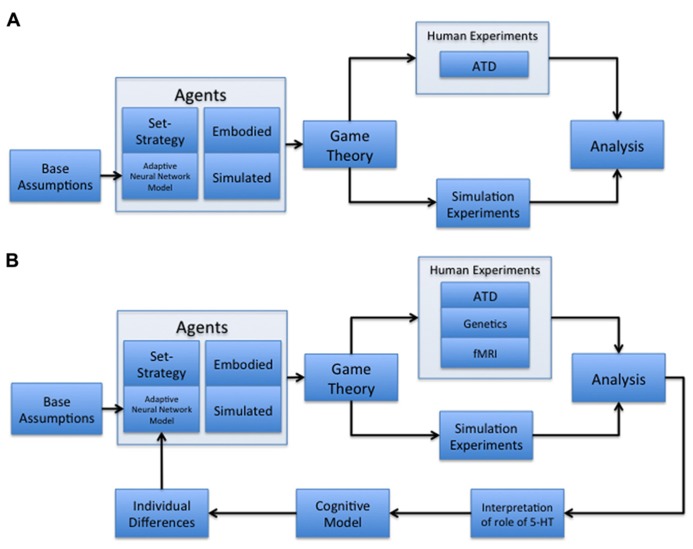
**Multidisciplinary paradigms. (A)** Multidisciplinary paradigm for investigating the role of serotonin in decision-making and behavior. The model begins with base assumptions regarding neuromodulation, which are then used to develop an adaptive neural network model of cost and reward assessment. This network is embedded in an agent acting as a player in a game theoretic environment, alongside control conditions with set-strategy agents. These agents are both embodied in robotic players and simulated in computer-based games. The agents are used in both human and simulation experiments to assess the adaptive network’s ability to behave naturally, as well as the human subjects’ reactions to the adaptive agent compared to set-strategy agents. Human subject experiments under this paradigm can include acute tryptophan depletion (ATD) manipulations. The results from human and simulation experiments are then processed to determine the validity of any hypotheses developed at the outset, in addition to the appearance of interesting emergent behavior. **(B)** Cyclic, multidisciplinary paradigm. This model is a modified version of (A) with an added iterative component, as well as the inclusion of both fMRI (Tanaka et al., 2007; Yoshida et al., 2010; Crockett et al., 2012; Seymour et al., 2012) and genetics (Bevilacqua and Goldman, 2011; Hyde et al., 2011; Loth et al., 2011) components of human experimentation. The addition of an iterative component allows the results of previous studies conducted under the paradigm to be analyzed for possible areas of improvement in the model, which are then committed as alterations. The new node represents the following three modifications: (1) make new interpretations as to the role of serotonin in human subject behavior; (2) develop a new cognitive model based on human subject behavior; and (3) modify the adaptive neural network to create agents that reflect observed individual differences in human subject behavior. This paradigm allows for a constantly improving neural network model that is increasingly more able to fit the demands of studying decision-making and behavior.

Our paradigm combines socioeconomic game theory with embodied models of learning and adaptive behavior (**Figure 1A**). In particular, we constructed our computational models to reflect 5-HT’s potential interplay with the expected cost of a decision (Daw et al., 2002; Cools et al., 2007), under the assumption that 5-HT, released by the DRN, can act as an opponent to dopamine. In this case, activation of the 5-HT system may cause an organism to be withdrawn or risk-averse, and the DA system causes the organism to be uninhibited or risk taking (Boureau and Dayan, 2010). Within the context of this paper, cost is defined as either the perceived loss of an expected payoff or harm from a potential threat, depending on the scope of the study it is used in. We will compare our present results using this paradigm with other studies, and discuss future steps that may lead to more accurate modeling of serotonin’s proposed role in assessing the tradeoff between cooperation and competition (**Figure 1B**).

### PARADIGM OVERVIEW

When considering the complex and highly varied behavior in decision-making during socioeconomic games (Lee, 2008a), adaptive agents (i.e., computer algorithms that can adjust their game playing behavior in response to the human player or to changes in the environment) provide a formidable means to engage human subjects that exceeds the abilities of set-strategy agents (i.e., algorithms that do not adjust their strategy over the course of a game) (Valluri, 2006). As a result of the dynamic nature of adaptive agents, there is a bidirectional influence between agent and player that is otherwise limited, as is the case for set-strategy agents. Additionally, adaptive agents are capable of changing their strategies over time, both between and during games. This allows for more organic player behavior that resembles interactions with a human subject. The advantage of using an adaptive agent over human subjects is that experimenters have greater control over how the agent performs, addressing a weakness commonly found in the highly variable and complex decision-making strategies of humans (Craig et al., 2013). Furthermore, adaptive agents themselves are a source of information, as it is possible to examine their internal processes and strategies that develop in response to the game environment. Adaptive agents work well in a simulation setting, running thousands of trials very quickly; however, it is often necessary to match simulations with comparable studies in human subjects to get a more complete picture of an agent’s behavior. In human studies, it is not only possible for human subjects to interact with a computer screen; these agents can also be embodied in robots for further investigation of human–robot interactions.

Embodied models have been shown to elicit strong reactions in humans (Breazeal and Scassellati, 2002; Kidd and Breazeal, 2004) and exhibit more natural and complex behavior than pure simulations (Krichmar and Edelman, 2002, 2005). For these reasons, embodied models provide a good platform for studying a wide range of cognitive functions. One previous study tested subjects’ engagement with robots, as compared to animated characters, in an experiment where subjects had to cooperate with, persuade, and assist the robot in the completion of various tasks (Kidd and Breazeal, 2004). Subjects found robots to be more credible, informative, and enjoyable to interact with compared to an animated character on a computer screen. Similar results were found by Wainer et al. (2007), further reinforcing the theory that robotic platforms are seen as more cognizant, helpful, and pleasant to work with as reported by subjects. This, in turn, has led other researchers to adopt robots as brain-based devices, because they provide a framework for understanding the interaction of simulated brain activity within a real environment. Furthermore, the embodied approach might serve as the foundation for the development of intelligent machines that adhere to neurobiological constraints (Krichmar and Edelman, 2002, 2005).

In order to further elucidate the role of serotonin and dopamine in decision-making, we have developed a multidisciplinary paradigm that incorporates embodied adaptive agents into interactive game environments (**Figure 1A**). Our general paradigm includes several key aspects, which we describe in detail below. In brief, we begin with base assumptions founded on previous studies that are used to construct an adaptive agent. That model, alongside set-strategy agents used in control conditions, are either embodied in a robotic platform (Agents: Embodied) or embedded in a computer interface (Agents: Simulated). Those agents are incorporated into a game theoretic environment in both human subject and simulation experiments. Human subject experiments include manipulation with ATD (Human Experiments: ATD). The data collected from these experiments are analyzed to either support or reject specific hypotheses about the role of serotonin in decision-making, or to create new models that explore the theories that emerge from the data.

### BASE ASSUMPTIONS

To start, we assume that serotonergic activity in the raphe nucleus is related to the expected cost of a decision. In this case, cost assessment can be related to harm or loss aversion (Doya, 2002; Millan, 2003; Cools et al., 2008; Crockett et al., 2009, 2012; Murphy et al., 2009; Tanaka et al., 2009; Takahashi et al., 2012), as well as risk in discounting reward (Schweighofer et al., 2008; Tanaka et al., 2009). These suppositions imply that decreased serotonergic activity would result in reduced harm aversion and reduced risk aversion in the decision-making process, along with some alteration of learning parameters influenced by cost. That is, the magnitude of cost in making decisions is perceived as less when serotonin levels are low. On the other hand, we assumed that DA activity was related to the expected reward of a decision (Schultz, 1997; Berridge and Robinson, 1998; McClure et al., 2003; Redgrave and Gurney, 2006). Under this assumption, reduced dopamine would result in reduced reward seeking behavior and manipulation of the learning parameters influenced by reward. In other words, the magnitude of reward in making decisions is perceived as less when dopamine levels are low. In this case, high serotonin levels have a strong influence over decisions resulting in less risk taking behavior.

Although controversial compared to other neuromodulators, evidence suggests that serotonergic neuromodulation features both tonic and phasic modes of activity (Briand et al., 2007; Schweimer and Ungless, 2010; Nakamura, 2013). The phasic mode is associated with transient bursts of neural activity from aversive stimuli (Schweimer and Ungless, 2010), whereas the tonic mode is represented by baseline activity (Briand et al., 2007) linked to reward magnitude assessment (Nakamura et al., 2008), outcome-based motivation (Bromberg-Martin et al., 2010), and behavioral regulation (Okada et al., 2011). Although not specific to serotonin, it has been suggested that phasic neuromodulation amplifies inhibitory connections and extrinsic inputs from the thalamus, whereas during tonic neuromodulation, intrinsic cortico-cortico connections are relatively higher (Kobayashi et al., 2000; Gu, 2002; Hasselmo and McGaughy, 2004; Lapish et al., 2006). Theoretical work has shown that this change in synaptic currents associated with phasic neuromodulation can produce a winner-take-all (WTA) network response (Krichmar, 2008). This WTA response is indicative of decisive, exploitive behavior. In addition, evidence has indicated that neuromodulatory activity is linked to increased plasticity (Gu, 2002; Fletcher and Chen, 2010; Shumake et al., 2010; Moran et al., 2013; Nakamura, 2013). Thus, phasic neuromodulation might increase future responses to salient stimuli.

The association between tonic/phasic neuromodulation and explore/exploit behavior was originally put forth by Aston-Jones and Cohen (2005) based on their observations of the noradrenergic system during studies with awake-behaving monkeys. Based on this and other empirical evidence, we have extended the exploration/exploitation idea to other neuromodulatory systems (Asher et al., 2010, 2012a; Zaldivar et al., 2010; Krichmar, 2013). Specifically, tonic levels of neuromodulation have been associated with distractible behavior and poor task performance, whereas phasic neuromodulation has been associated with attentiveness and good task performance (Aston-Jones and Cohen, 2005). Although tonic levels are associated with distractibility, this is a necessary component of the drive for exploration, or seeking out new sources of rewarding stimuli in an environment. The attentiveness associated with phasic neuromodulation is necessary for the ability to exploit a resource that has proven to be rewarding, and also to attend to salient stimuli in an environment.

These base assumptions have led us to develop models balancing cost and reward in decision-making through simulation of the neuromodulatory systems, which reflects neural activity in the brain and its resulting explorative and exploitive behaviors.

### ADAPTIVE AGENT MODELS

**FIGURE 2:**
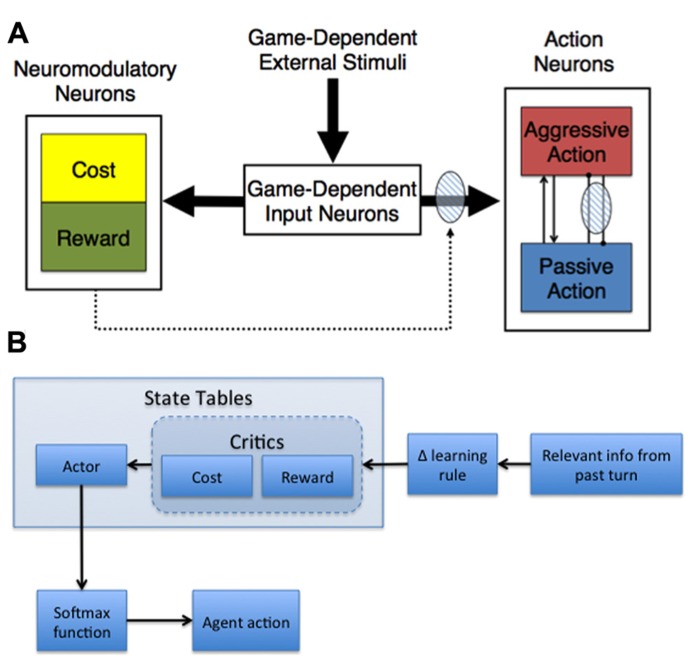
**Adaptive agent architectures. (A)** General neural network architecture for Hawk-Dove and Chicken studies. The thick arrows represent all-to-all connections. The dotted arrows with the shaded oval represent modulatory plastic connections. Within the Action Neurons region, neurons with excitatory reciprocal connections are represented as arrow-ended lines, and neurons with reciprocal inhibitory connections are represented as dot-ended lines overlaid by a shaded oval, which denotes plasticity. **(B)** Actor-Critic schematic. The behavior of the adaptive agent used in the Stag Hunt experiment (Craig et al., 2013) was controlled by an Actor-Critic model. The model was comprised of three state tables – Actor, Cost Critic, and Reward Critic – which were updated with relevant information from the most recent turn in the current game and then used to determine whether the agent should hunt stag or hare on the next turn. The payoff information from the last turn was combined with the cost/reward value associated with the current state (determined by the locations of tokens on the board) from each respective critic using a delta-learning rule. These modified values were then used to update the corresponding state in the Actor table, which was used in a SoftMax function to generate probabilities for hunting stag and hare. Those probabilities were then used to determine the agent’s action on the upcoming turn.

Given our base assumptions, we developed adaptive neural models capable of shaping action selection involved in decision-making (**Figure 2**). In general, these models made decisions based on their assessment of the expected cost and reward of actions, where cost was related to harm or loss aversion (Asher et al., 2010, 2012a; Zaldivar et al., 2010; Craig et al., 2013).

#### Neural network model

Our neural network model, which was used in Hawk-Dove and Chicken human robot interaction studies, simulated neuromodulation and plasticity based on environmental conditions, as well as previous experiences with cost and reward (Asher et al., 2010, 2012a; Zaldivar et al., 2010). The model was divided into three distinct neural areas: (1) Game-Dependent Input Neurons, (2) Action Neurons, and (3) Neuromodulatory Neurons (**Figure 2A**). The Game-Dependent Input Neurons, akin to sensory neurons, represented the possible environmental states the model could observe. The Action Neurons reflected the different choices the model could make in its environment. The Neuromodulatory Neurons featured Cost neurons, which represented serotonergic neuromodulation, and Reward neurons, which represented DA neuromodulation. The connections between the Game-Dependent Input Neurons and both the Neuromodulatory and Action Neurons were subject to neuromodulated synaptic plasticity. To model phasic neuromodulation’s effect on decision-making, neuromodulatory activity amplified the extrinsic excitatory connections from the Game-Dependent Input Neurons and the inhibitory connections from the opposing Action Neuron (**Figure 2A**).

The equation for activity of each of the Game-Dependent Input Neurons (*n*_i_) were computed as follows:

(1) $$n_{i}=\left\{ \begin{array}{c} b+noise;i=salient\,\;stimulus\\ noise;Otherwise \end{array}\right.$$

where *b* was a constant value dependent on the game played (*b* = 0.75 for the Hawk-Dove and *b* = 0.45 for the Chicken games described below), and *noise* represented neural noise, which was a random number between 0 and 0.25 drawn from a uniform distribution.

The neural activities for the action and neuromodulatory neurons were simulated by a mean firing rate neuron model, where the firing rate of each neuron ranged from 0 (quiescent) to 1 (maximal firing) on a continuous scale. The activity of both Action Neurons was based on their previous firing rates, plastic extrinsic excitatory input from the Game-Dependent Input Neurons, non-plastic intrinsic excitatory input from the opposing action neuron, and non-plastic intrinsic inhibitory input from the opposing action neuron (**Figure 2A**). In contrast, the activity of both Neuromodulatory Neurons was based on plastic extrinsic excitatory input from the Game-Dependent Input Neurons and previous cost/reward information reflected in the respective firing rates at the previous time step. The equation for the mean firing rate neuron model was:

(2) $$s_{i}(t)=\rho_{i}s_{i}(t-1)+\left(1-\rho_{i}\right)\left(\frac{1}{1+\exp^{\left(-5I_{i}\left(t\right)\right)}}\right)$$

where* t* was the current time step, *s*_i_ was the activation level of neuron *i*, *ρ*_i_was a constant set to 0.1 denoting the persistence of the neuron, and *I*_i_ was the synaptic input. The synaptic input of the neuron was based on pre-synaptic neural activity, the connection strength of the synapse, and the amount of neuromodulatory activity:

(3) $$I_{i}\,\left(t\right)=n\,o\,i\,s\,e+\underset{j}{\Sigma }\,n\,m\,\left(t-1\right)\,w_{i\,j}\,\left(t-1\right)\,s_{j}\,\left(t-1\right)$$

where *w*_ij_ was the synaptic weight from neuron *j* to neuron *i*, and *nm* was the level of neuromodulation, which was the combined average activity of the Cost and Reward neurons. The *noise* term represented neural input noise and was a random number between -0.5 and 0, drawn from a uniform distribution.

Phasic neuromodulation can have a strong effect on action selection and learning (Krichmar, 2008). During phasic neuromodulation, extrinsic excitatory synaptic projections from sensory systems and intrinsic inhibitory inputs are amplified relative to recurrent or excitatory intrinsic connections. In the model, the input (Game-Dependent Input Neurons) to Action neurons represented sensory connections and the inhibitory Action-to-Action neurons represented the intrinsic inhibitory connections. To simulate the effect of phasic neuromodulation, intrinsic inhibitory and sensory connections were amplified by setting *nm* in Equation 3 to ten times the combined average activity of the simulated Cost and Reward neurons. Otherwise, *nm* in Equation 3 was set to 1 for all other connections. In previous simulation studies and robotic experiments, this mechanism was shown to be effective in making the network exploitive when neuromodulation levels were high and exploratory when neuromodulation levels were low (Krichmar, 2008; Cox and Krichmar, 2009).

After the neural activities for the Action and Neuromodulatory Neurons were computed, a learning rule was applied to the plastic connections (projections from Game-Dependent Input Neurons) of the neural model. The learning rule depended on the current activity of the pre-synaptic neuron, the post-synaptic neuron, the overall activity of the modulatory neurons, and the cost/reward outcome from the game played:

(4) $$\Delta \,w_{i\,j}=\alpha^{*}\,n\,m\,\left(t-1\right)\,s_{j}\,\left(t-1\right)\,\;{\left(s_{j}\,\left(t-1\right)\right)}^{*}\,R$$

where *s*_j_ was the pre-synaptic neuron activity level, *s*_i_ was the post-synaptic neuron activity level, *nm* was the average activity of the Neuromodulatory Neurons, and *R* was the level of reinforcement based on payoff and cost (Equation 5). The pre-synaptic neuron (*s*_j_) in Equation 4 was the most active Game-Dependent Input Neuron (Equation 1). The post-synaptic neuron (*s*_i_) could be the most active Action neuron, the Cost neuron, or the Reward neuron. The level of reinforcement was given by:

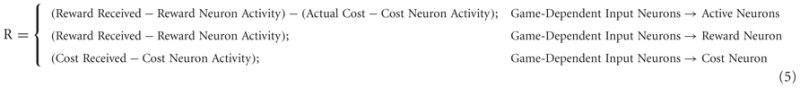



where the *Reward* *Received *and *Cost* *Received* were values determined by the positive and negative payoffs, respectively. The values were determined by a payoff matrix specific to the game being played (Asher et al., 2012a). Application of Equation 5 was based on the assumption that the Reward neuron activity predicted the reward of an upcoming action and the Cost neuron activity predicted the cost of that action. If the predictions were accurate, there would be little change in synaptic plasticity, whereas if the predictions were inaccurate, synaptic plasticity would occur (Equations 4–5).

#### Actor-critic model

In addition to the neural network described above, we have implemented more abstract adaptive agents based on our assumptions (**Figure 2B**). For example, a variation of the Actor-Critic model was used to simulate reward and cost assessment in a Stag-Hunt game (Craig et al., 2013). In general, the Actor-Critic model should abide by the tenets of game theory, learning to behave in such a way that maximizes gains and minimizes potential losses. Our model contained three state tables – one for the Reward Critic, Cost Critic, and Actor - that were comprised of a column for scalar weight values, similar to the plastic weights in the neural model described in the previous section, and several columns representing the state of the environment, akin to the sensory neurons in our neural model (see **Figure 2A**, Game-Dependent Input Neurons). The weights for the Cost and Reward Critics indicated the expected cost and expected reward values learned over time. For instance, state values in the Stag-Hunt game were related to the distances of the agent and the other players to potential rewarding stimuli. The weights in the Cost and Reward Critic state tables were governed by a delta rule for error prediction:

(6) δ⁢(t)=r⁡(t)+V⁢(s,t)−V⁢(s,t−1)

where *r*(*t*) was either the reward or cost at time *t*, *V*(*s, t*) was the Critic’s weight at state *s*, at time *t*, and *V*(*s*, *t*–1) was the Critic’s weight for the previous timestep. More specifically, the reward *r*(*t*) value corresponds to the agent’s expected value of their selected choice of rewarding stimuli at that timestep, and the cost *r*(*t*) is the negative of that value in the case that the expected reward was not fulfilled (i.e., perceived loss). However, other interpretations of cost are possible depending on the game being played. The delta value of Equation 6 was used to update the weights in the Reward and Cost critic tables at every timestep according to the following function:

(7) V⁢(s,t+1)=V⁢(s,t)+δ⁢(t)

The Actor weights were the likelihood to execute a particular action at a state and were updated by using the reward and cost information for that state. In the case that the model decided on choice 1 of two choices, the Actor weights were updated based on the following equation:

(8) $$\begin{array}{c} \begin{array}{c} V\,\left(c\,1,s,t+1\right)=V\,\left(c\,1,s,t\right)+1-P\,\left[c\,1\right]*\delta \,\left(t\right) \end{array}\\ V\,\left(c\,2,s,t+1\right)=V\,\left(c\,2,s,t\right)+1-P\,\left[c\,2\right]*\delta \,\left(t\right) \end{array}$$

*V*(*c*1, *s*, *t*) was the Actor’s state table value for deciding on choice 1 (of two possible actions) in state *s* at time *t*. Likewise, *V*(*c*2, *s*, *t*) was the Actor’s state table value for choice 2 in state *s* at time *t*. δ (t) was the delta value from both the Reward and Cost Critics. Thus, the Actor was updated based on the assessment of both the Cost and Reward Critics.The probabilities for selecting either choice 1 or choice 2 were decided using a SoftMax function:

(9) $$\begin{array}{c} P\,\left[c\,1\right]=\frac{e^{V\,\left(c\,1,s,t\right)}}{e^{V\,\left(c\,1,s,t\right)+e^{V\,\left(c\,2,s,t\right)}}}\\ P\,\left[c\,2\right]=1-P\,\left[c\,1\right] \end{array}$$

This implementation of the Actor Critic provided a cost-reward tradeoff mechanism for decision-making in game environments, analogous to the interplay between the DA and serotonergic neuromodulatory systems.

### GAME ENVIRONMENTS

In our experiments, we utilized both an adaptive neural network (**Figure 2A**) and an instantiation of the Actor-Critic model (**Figure 2B**) to investigate cost and reward in games of decision-making.

The adaptive neural network of **Figure 2A**, coupled with set-strategy models as controls, were both experimentally embodied as robotic agents and embedded in computer simulation within a game theoretic environment to investigate reciprocal social interactions depending on reward and cost assessment. For these experiments, we selected the game of Hawk-Dove, which is similar to the widely studied Prisoner’s Dilemma (Kiesler et al., 1996) but arguably more informative when studying a model of the serotonergic system’s role in cost assessment in competitive situations.

**FIGURE 3:**
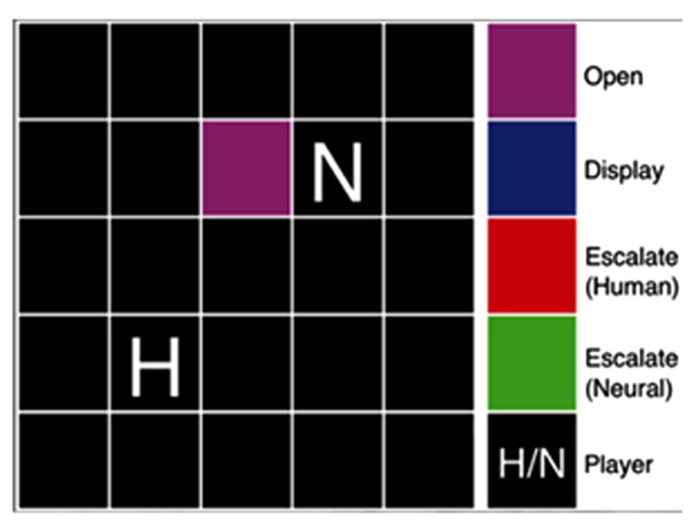
**Hawk-Dove game diagram.** The game board included a 5 × 5 grid of squares, upon which a territory was marked and the human and neural agent players were placed. The color of the territory reflected the state of the players’ actions. In the Hawk-Dove, two players must compete for a territory, deciding either to be submissive (display) or aggressive (escalate), avoiding or risking injury in hopes of a larger payoff, respectively. © 2012 IEEE. Reprinted, with permission, from Asher et al. (2012a).

Our version of Hawk-Dove (**Figure 3**) was played with an adaptive neural network model contesting over a resource with another player in an area referred to as the territory of interest (TOI) (Asher et al., 2010, 2012a; Zaldivar et al., 2010). The game started with each player and the TOI randomly placed inside an environment. In the Hawk-Dove game, each player needed to reach the TOI and choose between two actions: escalate (an aggressive, confrontational tactic) or display (a nonviolent, cooperative tactic). If both players chose to escalate, they fought, resulting in an injury or penalty, which could either be serious or mild. If only one player chose to escalate, then the escalating player received the total value of the TOI, and the other player received nothing. If both players chose to display, then there was a tie, and both players split the value of the TOI. Our variant of Hawk-Dove also modified the harshness of the environment in certain experimental conditions by increasing the likelihood of receiving a serious injury when escalating. Thus, players should strive to cooperate and minimize penalty from escalating by either alternating their actions or sharing the resource, which, at times, may result in conflict, as each player is attempting to secure the highest payoff.

**FIGURE 4:**
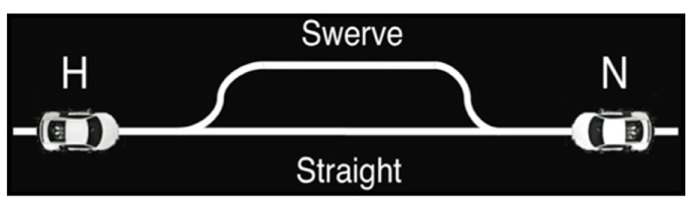
**Chicken game diagram.** Two toy cars, one driven by the human and one by the neural agent, were placed at opposite ends of a track. The cars started moving toward each other at the same speed and at the same time, at which point players must decide whether to conservatively swerve out of the way, but take a smaller payoff, or take the risk of a collision and continue straight ahead in hopes of a larger payoff. © 2012 IEEE. Reprinted, with permission, from Asher et al. (2012a).

Alongside Hawk-Dove, Chicken (Rapaport and Chammah, 1966) was used to investigate competitive situations in terms of expected costs and reward. Unlike Hawk-Dove in which players could decide to choose their action first or wait to see the other player’s decision, Chicken forced players to decide on an action quickly without knowledge of the opponent’s choice, as players do not know the decision their opponent has made until the outcome. In our version of Chicken (**Figure 4**), the human subject and the adaptive neural network model each controlled racecars, both heading toward each other on a single lane track (Asher et al., 2012a). If a player chose to swerve, that player relinquished the single lane track to the other player and received no reward, while the player who did not swerve received the maximum payoff. If both players swerved, they each received the minimum payoff. If neither player swerved, then the result was a severe head-on collision, the worst outcome for both players. Thus, the best outcome for a given player was to stay straight while the other player swerved. This created a situation in which each player, in an attempt to secure the best outcome, risked the worst scenario in terms of payoff.

**FIGURE 5:**
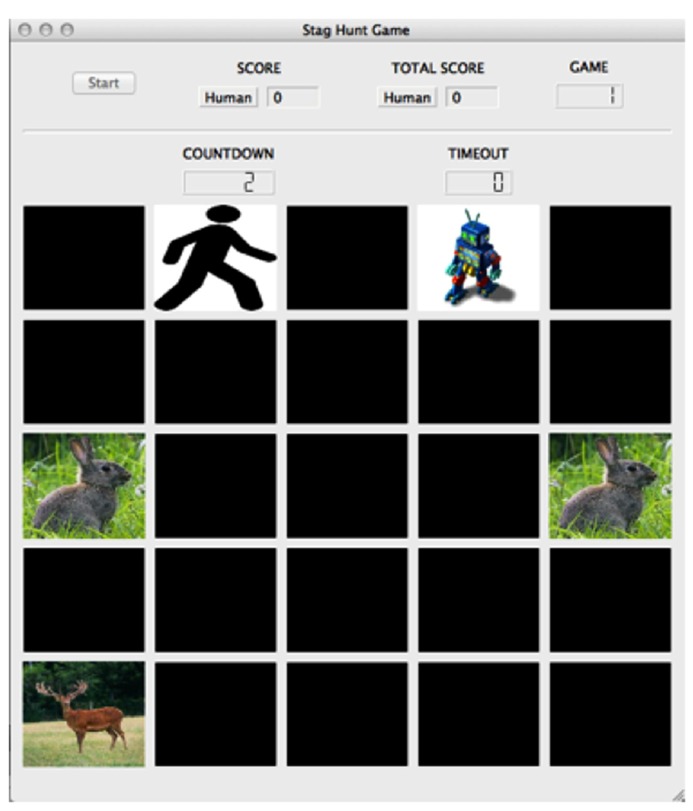
**Stag Hunt game environment**. The game board included a 5 × 5 grid of spaces upon which the player (stick figure image), agent (robot image), stag (stag image), and hare (hare image) tokens resided. The screen included a button to start the experiment, the subject’s score for the round, the subject’s overall score for the experiment, the game number, a countdown to the start of the game, and a counter monitoring the game’s timeout. In the game of Stag Hunt, two players attempt to hunt a low-payoff hare alone, or attempt to cooperate with the other player to hunt a large payoff stag. © 2013 by Adaptive Behavior. Reprinted by Permission of SAGE from Craig et al. (2013).

While games such as the Prisoner’s Dilemma, Hawk-Dove and Chicken are used to explore cost and reward assessment in competitive situations, the socioeconomic game known as the Stag Hunt is better suited to investigate cooperative situations and the formation of social contracts. Evidence suggests that neural responses are different when the social interaction is perceived to be cooperative versus competitive (Fleissbach et al., 2007). In Stag Hunt, two players must decide whether to cooperate with each other in order to hunt the high-payoff stag, or hunt a low-payoff hare individually (Skyrms, 2004). The risk involved with stag hunting is that both players must commit to hunting stag. If one player hunts stag while the other player hunts hare, the stag hunter is unable to catch the stag and receives no payoff. While the standard version of Stag Hunt is typically played as a simple stag or hare choice, we used a variant of the game, much like the one used by Yoshida et al. (2010), that incorporated a spatiotemporal component (**Figure 5**). The game was played on a computer-simulated 5 × 5 board, with tokens depicting the locations of the two players, the stag target, and the hare targets. Players moved toward the targets at the start of each game, with enforced token adjacency as a condition to catch them. Cooperation is crucial in Stag Hunt, as in order to obtain the highest payoff (stag capture) players must form social contracts to work together.

### TESTING THE ADAPTIVE MODELS IN GAME ENVIRONMENTS

Depending on the goal of the experiment in question, simulations can provide significant information about behavior development in an adaptive model. These experiments often consist of exhaustive model testing with various opponents, environmental conditions, and intrinsic model parameters resulting in various behaviors and strategies that the model may exhibit. With our model of cost and reward modulation (see Neural Network Model), we conducted simulation experiments that revealed that the model was capable of predicting upcoming costs and rewards (Asher et al., 2010; Zaldivar et al., 2010). This resulted in the evolution of mixed strategies that allowed the model to compete for resources, independent of the opponents’ actions. With our instantiation of an adaptive Actor-Critic model (see Actor-Critic Model) embedded in the Stag Hunt game, we found that this model developed suitable state tables to guide the agent in cost and reward prediction while playing against set-strategy agents (Craig et al., 2013). In both cases, the simulations showed that the adaptive model was sensitive to the other player’s strategy and the game environment. For example, when making decisions in the Stag Hunt, the model not only took into consideration its distance to the game tokens, but also the other player’s distance to tokens. These simulation experiments provide evidence that the base assumptions were a sufficient foundation for the model governing behavior. However, simulation experiments are only a small subset of the methods that can be utilized when studying an adaptive model’s behavior. It is also important to observe real human interaction with the model in an effort to assess the model’s ability to replicate natural behavior.

Following simulation, human subject experiments were performed to test the adaptive model’s performance against human players, as well as the subjects’ reactions to playing against both set-strategy and adaptive agents, and the influence of embodied agents on game play. Our first set of human subjects experiments involved ATD, the dietary manipulation described above that temporarily lowers serotonin levels in the central nervous system, resulting in decreased cooperation and lowered harm-aversion (Wood et al., 2006; Crockett et al., 2008). In these ATD experiments, two sessions (tryptophan-depleted and control) were performed on two separate days. For each session, healthy, adult subjects played the Hawk-Dove (**Figure 3**) and Chicken (**Figure 4**) games against adaptive agents both in simulation and embodied in physical robots. We measured changes in behavior associated with lowered levels of 5-HT throughout the interactions between human subjects and the robotic agent in the game environments.

In our next set of human subject experiments, the participants played the Stag Hunt game with various set-strategy and adaptive simulated agents. Subjects played games against each of five computer strategies, including an adaptive model. In each game, players navigated the game board on a computer (**Figure 5**), ending the game when either one of the players successfully captured a hare, or both players worked together to capture a stag. The adaptive agent was an instantiation of the Actor-Critic model that weighed cost and reward to make decisions in the environment in a manner much like the serotonergic and DA systems are thought to act in humans (Cools et al., 2010). Using these paradigms, we were able to study the ability for humans to cooperate with adaptive agents, as well as the extent of learning that takes place in the model when placed in an environment fostering cooperation.

Altogether, our multidisciplinary paradigm is one of many that are currently utilized in this field to explore the theorized role of the serotonergic system on behavior as related to cost assessment. The results from using this paradigm provide a balanced and informative procedure that incorporates both neuromodulation and behavior with current methods and technology, as described below.

## RESULTS OF OUR STUDIES CONDUCTED USING THIS MULTIDISCIPLINARY APPROACH

### ADAPTIVE NEURAL NETWORK PLAYING THE HAWK-DOVE GAME

We explored the research question of how the interplay between cost and reward would lead to appropriate decision-making under varying conditions in a game theoretic environment. To test this question, we modeled several predictions as to how the activity of a cost function leads to appropriate action selection in competitive and cooperative environments (Asher et al., 2010; Zaldivar et al., 2010). One such prediction was that the interaction between the simulated serotonergic neuromodulatory system, associated with the expected cost of a decision, and the simulated DA system, associated with the expected reward of a decision, would allow for appropriate decision-making in Hawk-Dove (see **Figure 2A** and Adaptive Agent Models). Our results verified this prediction, as the adaptive neural agent was more likely to escalate over the resource when activity of the reward system exceeded the activity of the cost system. Conversely, when the reward activity did not exceed the activity of cost, the adaptive neural agent displayed. One further prediction verified by our results was that the impairment of the serotonergic system would lead to perseverant, uncooperative behavior. A simulated lesion of the serotonergic system resulted in the adaptive neural agent almost always engaging in risk taking (aggressive) behavior, which was similar to the uncooperative behavior seen in human studies where serotonin levels were lowered via ATD while subjects played games such as Prisoner’s Dilemma and the Ultimatum game (Wood et al., 2006; Crockett et al., 2008). Altogether, our results are in agreement with the theoretical work proposed by Boureau and Dayan (2010), in which the influence of serotonergic and DA systems in generating an appropriate decision are sometimes in opposition.

### ATD AND EMBODIMENT IN HAWK-DOVE AND CHICKEN GAMES

To test the influence of embodiment and serotonin on decisions where there is a tradeoff between cooperation and competition, we conducted a study that included both embodied and simulated versions of adaptive agents along with manipulation of serotonin in human subjects. We used ATD to reveal the ways humans interacted with these agents in competitive situations via the Hawk-Dove and Chicken games (Asher et al., 2012a). Although we did not look at the ratio of plasma tryptophan to other large neutral amino acids, the differences between total blood plasma tryptophan levels with (5–8 μmol/L) and without (51–182 μmol/L) ATD were highly significant (*p* < 0.0005, Wilcoxon rank-sum test). Contrary to our expectations, we found that our subjects’ ability to assess cost when tryptophan-depleted was unchanged and that they were not more likely to cooperate with an adaptive embodied agent at the population level of analysis. Subjects responded equally strong to both the embodied and simulated adaptive agents, and tryptophan-depleted subjects did not show a significantly increased proportion of aggressive decisions (escalate) resulting from a decrease in cost assessment. Instead, we found that subjects significantly altered their strategy from Win-Stay-Lose-Shift (WSLS) against control adaptive agents, to Tit-For-Tat (T4T) against an aggressive version of the model, which is in agreement with previous studies (Wood et al., 2006; Crockett et al., 2008). We interpreted this result as subjects tending toward retaliatory behavior when confronted with agents that partook in risky behavior. This result was in agreement with those found by Crockett et al. (2008), which indicated that subjects under ATD tended to reject significantly more unfair offers in the Ultimatum game. The rejection of unfair offers in the Ultimatum game is similar to the retaliatory behavior observed in both the Hawk-Dove and Chicken games. Additionally, a type of motivational opponency was found in the dorsal and ventral regions of the striatum when subjects received costly punishment in the Ultimatum game (Crockett et al., 2013). Given that serotonin has been shown to have an inhibitory effect on the striatum (Di Cara et al., 2001), decreased 5-HT levels led to greater striatal activity. Similarly, subjects demonstrating retaliatory behavior under the effects of ATD would likely have shown increased dorsal striatal activity, a result that has been observed in previous work (de Quervain, 2004; Krämer et al., 2007; Strobel et al., 2011). Although we did not collect any brain imaging data, we would expect to see differences in striatal activity across our subjects correlating with their individual baseline levels of retaliatory behavior.

Although the small subject size (*n* = 8) may have contributed to the lack of significant differences in our measurements of both tryptophan-depletion vs. control conditions and embodied agent vs. simulation conditions, there is the possibility that differences between the conditions were masked by subgroups of subjects responding differently across conditions.

### COGNITIVE MODELING

To better understand our results at the individual subject level, we implemented a cognitive model to investigate potential behavioral differences in the subjects’ decision-making by examining their propensity to choose the aggressive action (escalate) in the Hawk-Dove game under the various conditions. Because these cognitive models use Bayesian inference to predict subject behavior based on many individual decisions, their predictions were not weakened by a small sample size.

To investigate how ATD and embodiment affected subjects’ decision-making in our previous work (see ATD and Embodiment in Hawk-Dove and Chicken Games), we implemented a cognitive model using hierarchical Bayesian inference. Hierarchical Bayesian inference has been shown to be a highly customizable and reliable way of exploring models of cognitive processes (Rouder et al., 2005; Lee, 2008b; Wetzels et al., 2010). In addition, Bayesian graphical models have been used to make inferences about the use of strategies such as WSLS or T4T from data consisting of sequences of choices from human subjects studies in N-armed bandit tasks, as well as other sequential decision-making tasks (Lee et al., 2011; Newell and Lee, 2011).

We used a hierarchical latent mixture model with Bayesian inference to analyze the individual differences in decision-making arising from alterations in serotonin levels and of agent embodiment (Asher et al., 2012b). The hierarchical attribute of these models allows for modifications to the parameters controlling cognitive processes across different individuals. We decided to use latent mixture models, as they allow for modeling completely different strategies across individuals. Formally, we recast the cognitive models as probabilistic graphical models and used Markov Chain Monte Carlo (MCMC) methods for computational Bayesian inference. By utilizing hierarchical latent mixture models, we addressed the question of how ATD and embodiment in the Hawk/Dove game could affect subjects’ decision to compete (i.e., choose the aggressive escalate action) or cooperate (i.e., choose the passive display action). We modeled the probability of escalating through a logistic model. The logit (Cramer, 2003) of the probability of escalating for each subject in each condition was assumed to follow a Gaussian distribution defined by its mean and variance (hyperparameters in the hierarchical model), with the mean modeled as the sum of the baseline level of escalating for the subject, and an additive effect associated with ATD or embodiment (Asher et al., 2012b).

**FIGURE 6:**
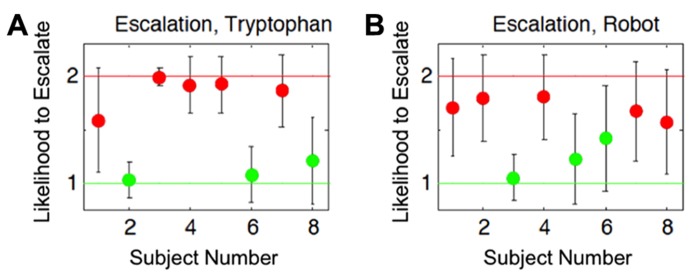
**Estimated group identities based on cognitive modeling results.** Both plots show each subject’s likelihood to choose the aggressive action (escalate) for the two different conditions. Red and green dots correspond to subjects that showed a respective increased or decreased probability to escalate from their baselines. Error bars show the 95% Bayesian confidence interval of the posterior mean. The *x*-axes indicate subject numbers, which correspond to the same subjects in the two plots. The *y*-axes show the Bayesian model’s mean output indicating group affiliation with respect to the subject’s likelihood to escalate, relative to their independently determined baselines. The *y*-axis value of 1 indicates a strong likelihood of decreasing choices to escalate relative to their baseline level for the conditions, whereas the value of 2 indicates a strong likelihood of increasing choices to escalate relative to their baseline level for the conditions. The group identities were estimated based on: **(A)** the influence tryptophan depletion had on subjects’ choices for aggressive actions (Escalation, Tryptophan), and **(B)** the influence an embodied agent had on subjects’ choices for aggressive actions (Escalation, Robot). Cognitive Science Conference and published in the Proceedings (COGSCI 2012, Sapporo, JP) from Asher et al. (2012b).

We showed that subjects separated into two distinct subgroups for the probability to choose the aggressive action (escalate) across the conditions (**Figure 6**). Our justification for this conclusion was based on the assumption that the effect of ATD/embodiment could vary across individuals as is reinforced by recent evidence suggesting that the effects of ATD give rise to individual differences across subjects (Krämer et al., 2011; Demoto et al., 2012; Seymour et al., 2012). The individual differences observed could either result in an increase or decrease in the likelihood of selecting an aggressive action. Alternatively, between the two subgroups, there existed a potential middle ground that was relatively unbiased on the scale of increased or decreased selection of aggressive actions, which is analogous to random behavior or the lack of influence from the experimental conditions (null hypothesis). No subjects fell within this middle ground for the conditions shown in **Figure 6**, further reinforcing a strong possibility for the existence of at least two subgroups within the subject population (*n* = 8). The results from this analysis yielded a differential influence on subjects stemming from lowered serotonin levels and of agent embodiment on individual decision-making in a competitive game (Asher et al., 2012b), which potentially implicates neural correlates in these individual differences.

To give a full account of the data, the hierarchical model was designed to address individual differences at two levels: the baseline level, which depends on the subjects inherent tendencies, and the additive level, which depends on the interaction between subjects natural tendencies and experimental conditions. In contrast to the results from our population analysis (see ATD and Embodiment in Hawk-Dove and Chicken Games), we found that clustering subjects into two opposing subgroups better represented the data. That is, one group of subjects, in concordance with expectation, had a higher probability to escalate under tryptophan depletion, but another had a lower probability to escalate in the tryptophan-depleted condition (**Figure 6**). Similarly, we found that two subgroups better predicted the rate of escalation when comparing responses to a robot versus responses to a computer simulation (**Figure 6**). The formation of these subgroups is not accounted for by the variance of the data in the population analysis (Asher et al., 2012a). We hypothesized that these subgroups may be typical of any given population of human subjects under these conditions and that future studies should take individual variation into consideration. This framework for evaluating cognition offers a comprehensive approach for modeling individual differences in cognitive strategies (Lee, 2008b; Lee et al., 2011).

### HUMANS PLAYING STAG HUNT WITH SIMULATED ADAPTIVE AGENTS

In our recent study using the Stag Hunt game, we investigated the variance in behavior of human subjects while playing Stag Hunt against adaptive (cost/reward learning) and set-strategy agents, with the intent of finding a stronger response evoked by adaptive over set-strategy. We found that adaptive agents, controlled by an Actor-Critic model (see **Figure 2B** and Adaptive Agent Models), caused subjects to invest more time and effort into game play than set-strategy agents (Craig et al., 2013). The strategy of the adaptive agent was formed by taking into consideration the reward and costs of its decisions, much like the theorized roles of the DA and serotonergic systems, while the four set-strategy agents conformed to the following tactics: (1) always hunt hare, (2) always hunt stag, (3) act randomly, and (4) WSLS. During games with an adaptive agent, human subjects took significantly longer to make a move than when playing against the other agents. Specifically in games in which the subject did not receive a payoff (i.e., the subject lost the game), subjects took a significantly longer path across the board to their endgame position than in all other tested set-strategy conditions. These findings indicate that playing against adaptive agents correlated with more effort spent on the subject’s part while making decisions. Moreover, it appears that subjects might have been trying to guide the adaptive agents toward stags; such a strategy would suggest that, purely through experience, subjects became aware of the fact that the adaptive agent, unlike the set-strategy agents, could be influenced. The increased time and effort exerted by the subjects in the adaptive condition from our Stag Hunt experiment, may be related to increased neural activity seen in other Stag Hunt studies (Yoshida et al., 2010). Using fMRI while subjects played the Stag Hunt, Yoshida et al. (2010) observed increased activity in rostral medial and dorsolateral prefrontal cortices when subjects played a more sophisticated (adapting) agent, areas that indicate planning and mentalization.

Similar to our findings with the Hawk-Dove game, the Stag Hunt study also highlighted subject variation when playing games of decision-making. When assessing the ratio of stag-to-hare captures, playing against an adaptive agent appeared to evoke different equilibriums of hunt decisions in individual subjects. It appears that, much like the Hawk-Dove results (Asher et al., 2012b), over half of analyzed subjects became either strongly cooperative or strongly competitive when playing the adaptive agent. Overall, these results showed that adaptive agents are able to evoke complex behavioral responses in human subjects that may vary depending on individual subject differences. This is useful when studying decision-making and also offers control over agent behavior that would not have been possible in human-human studies. While ATD was not performed in the Stag Hunt experiment, it is possible that the grouping of these subjects also resulted from individual differences, such as genetic polymorphisms related to the serotonergic system (Bevilacqua and Goldman, 2011; Hyde et al., 2011; Loth et al., 2011) leading to changes in cost/reward assessment.

## FUTURE DIRECTIONS USING A CYCLIC, MULTIDISCIPLINARY PARADIGM

In an attempt to more accurately model serotonin’s theorized influence on decision-making, we suggest that future experiments improve upon the approach illustrated in **Figure 1A** with the addition of an iterative component (**Figure 1B**). Such a cyclic paradigm would have the following components: (1) the development of an embodied neural model to support socioeconomic game studies; (2) an experimental protocol in which subject behavior and neural correlates of decision-making can be probed and categorized; (3) the design of an improved neural model that captures the neuromodulatory influences and individual variation of decision-making in socioeconomic games, to be used in subsequent experiments; and (4) the deployment of a population of models with varying phenotypes to be used in subsequent socioeconomic game studies. Components (3) and (4) allow the paradigm to run cyclically, thereby improving the paradigm through analysis and incorporation of past results. In this cyclic, multidisciplinary paradigm, we amend our previous multidisciplinary paradigm with a feedback loop that: (1) makes new interpretations for the role of serotonin in subject behavior; (2) develops a new cognitive model based on subject behavior; and (3) modifies the adaptive neural network to construct agents that capture individual behavioral differences demonstrated by subjects. These modifications are performed with the intention of refining the adaptive neural network’s performance, making its behavior more natural and human-like. After each cycle, the experimental paradigm improves to better suit the purposes of the task (e.g., stronger decision-making/modeling of neuromodulation), while holding constant the general framework of testing (e.g., game theoretic environment, simulation/human experimentation, etc.).

While the proposed paradigm is intended to improve the field of modeling decision neuroscience, our current models are rather abstract and would benefit from the incorporation of additional empirical data collected from the mammalian brain in neuroimaging and neurophysiological studies. Functional data from neuroimaging studies can help our models become more biologically realistic by revealing the specific brain areas active during select behaviors. Single unit recording studies in animals dictate the more granular neural behavior within each modeled brain region. Together, empirical data provides the base assumptions that guide our models computational neural behavior and architecture, making them more biologically realistic. Improved biological plausibility can, in turn, increase the efficacy of theoretical predictions made by our models, resulting in better theories to be tested through future neurophysiological and neuroimaging experiments.

Single unit recording studies in animals provide a critical component to computational modeling, as physical data is essential for developing base assumptions and confirming predictions made by models. For instance, phasic and tonic serotonergic activity in monkeys and rats has been associated with components of cost and reward processing (Nakamura et al., 2008; Bromberg-Martin et al., 2010; Schweimer and Ungless, 2010; Okada et al., 2011; see Base Assumptions). In future work, our base assumptions could more accurately incorporate the dynamics of both phasic and tonic serotonergic activity to improve the biological plausibility of our models. This could lead to better predictions about the dynamics between phasic and tonic serotonergic neuromodulation and their impact on cost and reward processing. Thus, where computational models inevitably rely on empirical data to make predictions about neuromodulatory influence over biological behavior, their predictions can provide theoretical evidence for future experiments.

Empirical data from neuroimaging studies provide a relationship between brain activity and behavior that can be used as the foundation for biological plausibility in a computational model. For example, fMRI has been used to determine the relationship between brain regions innervated by serotonin and behaviors involved with reward prediction (Tanaka et al., 2007), the perceived value of reward (Seymour et al., 2012), and the association of serotonin with reactive aggression under certain circumstances (Krämer et al., 2011), amongst other behaviors. The tasks and behavioral results are comparable to studies conducted using the initial paradigm outlined in this paper (see **Figure 1A**), but they include the relationship between serotonergic brain regions and the different behaviors. For example, in future models, we plan to implement the serotonergic influence over the striatum to obtain theoretical evidence for reward prediction based on different levels of serotonin, and its resulting effect on decision-making (Tanaka et al., 2007; Seymour et al., 2012). This empirical data could be included into future computational models leading to more diverse and organic model behavior, which could also aid in the development of a better theoretical understanding of the underlying relationship between serotonergic brain regions and their associated behaviors. While fMRI is incorporated into our cyclic, multidisciplinary paradigm (**Figure 1B**), results from neuroimaging studies outside of our paradigm remain an integral part of the foundation for biological plausibility in our models, and in turn, increases the efficacy of our paradigm.

In addition to these sources of empirical evidence, theoretical data from other biologically realistic models and neurally inspired robotic agents can contribute to the biological plausibility of our models and serve as a basis for further empirical investigation. For example, incorporating more biophysically detailed models of DA and serotonergic neuromodulation, such as (Chorley and Seth, 2011; Wong-Lin et al., 2012; Avery et al., 2013; Cano-Colino et al., 2013), may be informative. The temporal dynamics of these models, as well as the neuroanatomical pathways that they include, would be of interest when coupled with subject interactions.

Embodiment is a key element to the paradigm we are promoting, and these human robot interaction experiments may not only evoke strong responses in subjects, but they may also inform the development of future neurorobots. Embodied models using robotic platforms have provided clues as to how neuromodulation can give rise to adaptive behavior in biological systems (Krichmar, 2013; Luciw et al., 2013). In one such experiment, using an actor-critic model featuring a reinforcement learning algorithm allowed a biped NAO robot to develop locomotion and adjust its gait to different conditions (Li et al., 2013). These studies provide theoretical evidence for how adaptive behavior could develop in a biological system and suggest how this could be applied to a robotic system. Similarly, a recent biologically plausible model in simulation was able to develop motivated behavior by implementing an interplay between aversive and appetitive stimuli, which induced activity in their simulated serotonergic and DA brain regions, respectively (Weng et al., 2013). By closing the loop between brain, body, and environment, these embodied systems demonstrate how neuromodulators, such as dopamine and serotonin, can influence action selection and decision-making.

It is important to emphasize that the models used in our paradigm serve as a venue for investigating the influence of serotonin in motivational systems for robots and other autonomous systems. Future iterations of research through our paradigm could modify our model to increase the accuracy and scope of its biological representation. In contrast to other similar models that associate serotonin with decision-making (Daw et al., 2002; Doya, 2002; Weng et al., 2013), we modeled how phasic serotonergic neuromodulation could influence an autonomous agent’s behavior in a game theoretic environment (Asher et al., 2010, 2012a; Zaldivar et al., 2010). However, this abstraction is limited in extrapolating the role serotonin plays in decision-making in other environments because while game theory is a good tool for investigating decision-making, it explicitly places numerical value on the cost and reward elements of decision-making. In contrast, other biologically plausible models of neuromodulation and behavior have linked the value of a decision to novelty (Bolado-Gomez and Gurney, 2013; Krichmar, 2013), curiosity (Luciw et al., 2013), and uncertainty (Krichmar, 2013) in environments void of game theory. Krichmar (2013), built upon our work with a biologically plausible model of neuromodulation and behavior consisting of acetylcholine/norepinephrine (novelty), serotonin (withdrawal and harm aversion), and dopamine (invigoration and risk-taking) systems, in an autonomous robot that demonstrated anxious and curious states associated with rodent behavior. This work was able to use this expanded model to show that high levels of serotonin caused withdrawn behavior, while low levels of serotonin, in combination with high levels of dopamine, brought about excessive exploratory behavior. Additionally, top-down signals from the frontal cortex to the raphe nucleus were found to be critical for coping with stressful events. In the pursuit of more biologically accurate models of behavior and decision-making, biological neuronal modeling allows for studying hypotheses about serotonin’s involvement in behavior and learning that would otherwise be empirically difficult to test. Furthermore, predictions from such embodiment studies could motivate the design and scope of new animal studies.

Utilizing a cyclic paradigm lends itself especially well to studies that incorporate embodiment, as the internal mechanisms governing embodied models are constantly being updated to improve their behavior during interactions with human subjects. The evidence that human subjects are more likely to treat robotic platforms similarly to other humans rather than computer simulations (Breazeal and Scassellati, 2002; Kidd and Breazeal, 2004; Asher et al., 2012b) suggests that there are larger social expectations placed on embodied agents. Additionally, human subjects have been shown to report embodied agents as more helpful and aware when compared to simulated agents (Wainer et al., 2007), further justifying their use in studying human behavior and decision-making. Since the iteratively improved adaptive agents in our paradigm are modified to better simulate neuromodulatory influence and behave increasingly more like human subjects, their embodiment could lead to more robust decision-making and social interactions, which in turn leads to more compelling and informative human-robot interaction studies and better predictions about serotonergic and DA influence over behavior. Results of these studies ultimately lead to improved adaptive models situated in robots, which have value in a wide variety of applications (e.g., medical, commercial, industrial, etc.).

While implementing adaptive agents into robotic platforms is a promising venture for future study, past experiments have revealed individual differences between subjects that warrant the investigation of genetic sources. Because the results from our Hawk-Dove and Stag-Hunt experiments showed individual variation in game play, genetic screening for polymorphisms in human subjects could provide a venue for studying serotonin’s role in this variation. Several groups have suggested that individual differences in behavior are influenced by genetic polymorphisms related to serotonin signaling (Bevilacqua and Goldman, 2011; Hyde et al., 2011; Loth et al., 2011). Within this cyclic paradigm, one such proposed study could incorporate our current adaptive agents used to play cooperative (Stag Hunt) and competitive (Hawk-Dove and Chicken) games with a random sampling of human subjects, who would be screened for polymorphisms related to serotonergic function (e.g., 5-HTTLPR) (Homberg and Lesch, 2011). From the data analysis of these genetic polymorphism experiments, genetic-dependent diversity could be integrated over the neuromodulatory function of our adaptive agents, and any additional experiments necessitated by the predictions that emerged from the previous iteration through the paradigm would be conducted (**Figure 1B**). These experiments could allow for the generation of new hypotheses leading to predictions about the genetic variation in serotonergic neuromodulation and its ties to motivated human behavior. Ultimately, the predictions might help shape the next generation of empirical studies.

Though it is important to utilize new techniques such as genetic screening to better understand the role of serotonin in decision-making, a primary benefit to our paradigm is its incorporation of theoretical predictions from past work into future studies. Previously, we found that the concept of two opposing subgroups (**Figure 6**) best described the subjects’ behavior in the Hawk-Dove game. This theoretical data could be applied to the next generation of adaptive model (via the iterative component of **Figure 1B**) through additional assumptions or constraints of serotonergic neuromodulation. These new assumptions lead to better predictions about the diversity in behavior resulting from serotonergic manipulation. As another example, from the Stag Hunt human subject experiment, we discovered the tendency for adaptive agents to move counterintuitively when subject behavior was erratic. In a second iteration of experiments, we could improve upon this model by utilizing a top-down mechanism founded in neuromodulation to converge behavior in the face of seemingly random influence. By using cognitive models, we can create behavioral phenotypes in future models to match the potential individual differences that arise in any given population. These are a couple of examples of how this cyclic paradigm would help our adaptive models and ultimately our understanding of neuromodulatory influence over behavior in decision-making.

In terms of potential clinical application, the proposed paradigm may help illuminate components of brain disorders associated with abnormal serotonergic function. Serotonin has been implicated in a variety of neuropsychiatric conditions including bipolar disorder (Robinson et al., 2009), antisocial personality disorder (Deakin, 2003), anxiety disorder (Heisler et al., 1998; Lowry et al., 2008), and affective disorder (Lowry et al., 2008). Because serotonin is strongly involved in these neuropsychiatric diseases, many frequently prescribed antidepressant and anti-anxiety medications target serotonin receptors. However, due to the complex physiological action of serotonin, it is difficult not only to gage the effectiveness of these psychiatric drugs, but also to isolate the neural pathways relevant to the serotonergic regulation of these disorders. Our work modeled the theorized influence serotonin has on decision-making in the context of cost assessment, which was accomplished by simulated lesions of the cost assessment region of the model (Asher et al., 2010, 2012a; Zaldivar et al., 2010). This resulted in an increase in impulsivity that could possibly be extrapolated to deficits in learning associated with these neuropsychiatric disorders. The Stag-Hunt, which focused on cooperation, may be applicable in the study of social disorders such as autism. Manipulations of computational models could also mimic such disorders, which would lead to predictions regarding the neural correlates of the disorder and could possibly warrant drug or therapy experimentation that tests the model’s predictions.

Thus, the cyclic, multidisciplinary paradigm provides a strong approach toward making predictions about the neurobiology that ties serotonin to motivated behavior. As we continue to explore serotonin and its role in decision-making, future studies should consider applying this paradigm in order to accommodate the complex behavior that accompanies the activity of the serotonergic system. Adaptive neural models situated in a game theoretic environment utilized in both human and simulation experiments, accompanied with analysis that leads to an upgraded model for future use, is a strategy that lends itself to the production of valuable research in the fields of neuromodulation, behavior, technology, and neuropsychiatry.

## Conflict of Interest Statement

The authors declare that the research was conducted in the absence of any commercial or financial relationships that could be construed as a potential conflict of interest.
